# Acute Neurotoxicity of Antisense Oligonucleotides After Intracerebroventricular Injection Into Mouse Brain Can Be Predicted from Sequence Features

**DOI:** 10.1089/nat.2021.0071

**Published:** 2022-06-01

**Authors:** Peter H. Hagedorn, Jeffrey M. Brown, Amy Easton, Maria Pierdomenico, Kelli Jones, Richard E. Olson, Stephen E. Mercer, Dong Li, James Loy, Anja M. Høg, Marianne L. Jensen, Martin Gill, Angela M. Cacace

**Affiliations:** ^1^ Roche Pharma Research and Early Development, Therapeutic Modalities, Roche Innovation Center Copenhagen, Hørsholm, Denmark.; ^2^ Bristol Myers Squibb Research and Development, Princeton, New Jersey, USA.

**Keywords:** neurotoxicity, antisense oligonucleotides, intracerebroventricular dosing, calcium oscillations, sequence design algorithm

## Abstract

Antisense oligonucleotides are a relatively new therapeutic modality and safety evaluation is still a developing area of research. We have observed that some oligonucleotides can produce acute, nonhybridization dependent, neurobehavioral side effects after intracerebroventricular (ICV) dosing in mice. In this study, we use a combination of *in vitro*, *in vivo*, and bioinformatics approaches to identify a sequence design algorithm, which can reduce the number of acutely toxic molecules synthesized and tested in mice. We find a cellular assay measuring spontaneous calcium oscillations in neuronal cells can predict the behavioral side effects after ICV dosing, and may provide a mechanistic explanation for these observations. We identify sequence features that are overrepresented or underrepresented among oligonucleotides causing these reductions in calcium oscillations. A weighted linear combination of the five most informative sequence features predicts the outcome of ICV dosing with >80% accuracy. From this, we develop a bioinformatics tool that allows oligonucleotide designs with acceptable acute neurotoxic potential to be identified, thereby reducing the number of toxic molecules entering drug discovery pipelines. The informative sequence features we identified also suggest areas in which to focus future medicinal chemistry efforts.

## Introduction

Antisense oligonucleotides (ASOs) are being developed as therapeutics for disorders of the central nervous system (CNS) [1]. Due to the lack of blood–brain barrier penetrance of these large, negatively charged molecules, they must be injected directly into the cerebrospinal fluid to achieve effective target engagement in the brain or spinal cord in patients. Although nusinersen, an ASO approved for the treatment of spinal muscular atrophy, is efficacious and safe given intrathecally [2], safety assessment for ASOs is still a developing area of research. Mouse models are often used to assess safety and therapeutic utility, and to establish pharmacodynamic and pharmacokinetic parameters of ASOs under development [3].

A common method for evaluating *in vivo* activity of ASOs preclinically is by direct bolus injection into the cerebral lateral ventricles of mice [4,5]. We have observed that many ASOs are safely delivered to mice, while others are poorly tolerated. In this study, we sought to investigate properties of the ASOs, which might lead to acute CNS side effects, and to identify bioinformatics methods to eliminate such ASOs from our drug discovery pipeline.

ASOs are short, chemically modified oligonucleotides, designed to bind by Watson-Crick base pairing to perfectly complementary regions in RNA targets of interest, thereby modulating their function [6]. Besides binding to the intended RNA target, they may also bind imperfectly to only partially complementary regions in RNA, or to proteins, and through these unintended interactions elicit off-target effects [7]. Such off-target effects may potentially lead to adverse effects and toxicity. For example, it is well known that adverse effects of ASOs dosed systemically can include liver and renal toxicity. In the case of hepatotoxicity, the hepatocellular injury induced by some ASOs can be measured as elevations of alanine-aminotransferase levels in serum 3–15 days after first dose in mice [8].

Cellular assays of hepatotoxicity and cytotoxicity have been developed to evaluate this hepatotoxic potential of ASOs before dosing in mice [9–11]. The molecular mechanism has been linked to the endogenous ribonuclease H1 enzyme with both RNA hybridization-dependent [12,13] and hybridization-independent pathways [14]. In particular, some ASOs with sugar modifications conferring higher binding affinity, such as locked nucleic acids (LNAs) or constrained ethyls (cETs) [6], have been reported as having high hepatotoxic potentials [15]. However, a broad range of sequence features of ASOs can be associated with their hepatotoxic potential [16], and a systematic analysis of such features has allowed machine learning models to be trained to predict the hepatotoxic potential of LNA-modified ASOs [17].

Taken together, the hepatotoxic potential of ASOs can be controlled *in silico* as well as *in vitro*, before dosing systemically in mice. In comparison, potential toxic effects of ASOs after intracerebroventricular (ICV) dosing in mice have only been sporadically reported to date. In the scientific literature, to our knowledge, only unintended activation of astroglial or microglial responses or inflammation have been reported for some ASOs [18,19].

However, neurofunctional assessment of ASOs in rodents to evaluate potential acute effects has been recommended by the Oligonucleotide Safety Working Group [20], and an approach to assess motor function of rats 3 h after intrathecal administration of ASOs has previously been described [21]. In the patent literature, results of such neurofunctional assessments of ASOs with deoxy-, cET-, or 2′-O-methoxyethyl modifications to the sugar ring, have been reported for mice and rats and for both intrathecal and ICV administration routes [22–24]. For some of these ASOs, the acute tolerability behavior was reported to be impacted.

In this study, we report on the systematic assessment and scoring of acute tolerability behavior of mice for 1 h after ICV bolus injection of 148 different LNA-modified ASOs with full phosphorothioate (PS) backbone. We find that about 40% of the ASOs evaluated have moderate to severe acute tolerability observations that preclude further development.

Certain sequence features, most prominently the number and position of guanine (G) nucleotides relative to the 3′-end of the ASO, which increase the toxic potential of the ASO, and the number of adenine nucleotides, which decrease it, can be summarized as a sequence score and used to predict the acute neurotoxic potential with >80% accuracy. These sequence features were identified in a much larger set of 1,645 LNA-modified PS ASOs evaluated for changes in spontaneous calcium oscillations when incubated with neuronal cells, since this cellular assay was found to accurately predict the acute neurotoxic potential in mice. Elimination of acutely toxic ASOs early in a program allows for more robust preclinical efficacy studies, improves animal welfare practice, and reduces safety concerns should these side effects translate to higher species.

## Materials and Methods

### Oligonucleotide synthesis and purification

Single-stranded DNA oligonucleotides with complete PS backbones and LNA-modified flanks were synthesized on a MerMade 192 × synthesizer (Bioautomation, TX, USA) following standard phosphoramidite protocols. The final 50-dimethoxytrityl (DMT) group was left on the oligonucleotide for later use as lipophilic handle and chromatographic retention probe. The oligonucleotides were cleaved from the controlled pore glass solid support by means of aqueous ammonia and subsequently deprotected at 65°C for 5 h. The oligonucleotides were purified by solid phase extraction in TOP cartridges (Agilent Technologies, Glostrup, Denmark) using the DMT group, after which the DMT group was removed. As the last purification step, the oligonucleotides were eluted from the cartridge and evaporated to dryness.

The oligonucleotides were dissolved in sterile 0.9% saline solution, and the concentration of oligonucleotide in solution confirmed by calculating the Beer-Lambert extinction coefficient and measuring ultraviolet absorbance. Oligonucleotide identity and purity were validated by reversed-phase ultra-performance liquid chromatography coupled to mass spectrometry. Nucleobase sequences and chemical modification patterns for all synthesized oligonucleotides are available in Supplementary Table S1.

### Mouse behavioral studies and assessment of acute tolerability

Adult C57BL/6J female mice weighing between 20 and 30 g (Jackson Laboratories, Bar Harbor, ME) were housed with 3 to 4 mice per cage. Animals were held in colony rooms maintained at constant temperature (21 ± 2°C) and humidity (50 ± 10%) and illuminated for 12 h per day (lights on at 06:00 h). Behavioral studies were conducted between 07:00 and 15:00 h. Animals were maintained in accordance with the guidelines of the Animal Care and Use Committee of the Bristol Myers Squibb Company, and the “Guide for Care and Use of Laboratory Animals” published by the National Institutes of Health. Research protocols were approved by the Bristol Myers Squibb Company Animal Care and Use Committee.

ICV, freehand injections of ASO were performed using a Hamilton microsyringe fitted with a 27- or 30-gauge needle [4]. The needle was equipped with a polyethylene guard at 2.5 mm from the tip to limit its penetration into the brain. Mice were anesthetized using isoflurane anesthetic (1.5%–4%). The mouse was gently restrained on a flat surface and the needle tip was then inserted through the scalp and the skull, about 1 mm lateral and 0.5 mm posterior to bregma.

Once the needle was positioned, 100 μg ASO was given in a volume of 5 μL in 0.9% saline vehicle and injected into the right (or left) lateral ventricle over 20–30 s (*n* = 4–6 mice per treatment group). The needle was left in place for 10 s before removal. This procedure required no surgery or incision. Animals were warmed on heating pads until they recovered from the procedure.

For 1 h following the single bolus injection of ASO, animals were observed for behavioral side effects using a modified functional observational battery, as recommended by the Oligonucleotide Safety Working Group [20].

The severity of side effects was scored using a tolerability scale divided into five neurobehavioral categories: (1) hyperactivity, (2) decreased activity and arousal, (3) motor dysfunction/ataxia, (4) abnormal posture and breathing, and (5) tremor/convulsions. Each category was scored on a scale of 0 to 4. Scores for each category were summed to a final acute tolerability score going from 0 (no side effects) to 20 (severe signs in all categories, including convulsions resulting in euthanasia). The acute tolerability scores for each mouse were averaged to produce a representative score for each treatment group. The average acute tolerability scores for all ASOs dosed in mice are available in Supplementary Table S1.

### Measurement of spontaneous calcium oscillations in primary cortical neurons

Primary cortical neurons were prepared from Sprague-Dawley rat embryos on embryonic day 19. Cells were plated with 25,000 cells/well onto 384-well poly-D-lysine-coated fluorescent imaging plate reader (FLIPR) plates (Greiner Bio-One) in 25 μL/well neurobasal media containing B27 supplement and 2 mM glutamine. Cells were grown for 11 days at 37°C in 5% CO_2_ and fed with 25 μL of additional media on days 4 and 8. On the day of the experiment, media was removed from the plate and the cells were washed once with 50 μL/well of 37°C assay buffer (Hank's Balanced Salt Solution with 2 mM CaCl_2_ and 10 mM HEPES (4-(2-hydroxyethyl)-1-piperazineethanesulfonic acid) pH 7.4). To evaluate the role of N-methyl-D-aspartate (NMDA) and non-NMDA receptors, neuronal spontaneous calcium oscillations were measured under two conditions, in the presence and absence of 1 mM MgCl_2_, as described previously [25].

Cells were loaded with a cell permeant fluorescent calcium indicator dye, fluo-4 AM (Life Technologies). Fluo-4 AM was prepared at 2.5 mm in dimethyl sulfoxide containing 20% plutonic F-127 and then diluted 1:1,000 in assay buffer. Cells were incubated for 1 h with 20 μL of 2.5 mM fluo-4 AM at 37°C in 5% CO_2_. After 1 h, 20 μL of room temperature assay buffer was added and the cells were allowed to equilibrate to room temperature for 10 additional minutes and placed in the FLIPR.

For studies confirming the α-amino-3-hydroxy-5-methyl-4-isoxazolepropionic acid (AMPA) receptor dependency of calcium oscillations in Mg^2+^ containing buffer (NMDA-independent), the total number of peaks in a 3-min period were calculated for vehicle and cyanquixaline (CNQX)-treated cells. Data were expressed as percent of vehicle-treated control.

For studies used to identify ASOs for advancement to evaluation in mice, a different system to evaluate calcium oscillations was used. This system was designed to capture both increases and decreases in oscillation frequency following ASO treatment. First, baseline signal of intracellular calcium levels was read for 100 s (1 reading/s) before the addition of ASO. ASOs were added with a 384-well head in the FLIPR in 20 μL of assay buffer at 75 μM for a final concentration of 25 μM. FLIPR signal was read for an additional 200 s (1 reading/s) after the addition of ASO. After 5 min, a second read of FLIPR signal was conducted for 300 s (1 reading/s) to allow for additional data capture.

Spike amplitude and frequency of calcium oscillations were calculated from FLIPR signal reads. An average control value was established by measuring the average FLIPR spike amplitude signal over a 300 s read for nontreated wells. A scoring system was developed where a score of 1 was given for each 1 s read where signal increase was >50% of the average control amplitude value. A score of 0 was given for each 1 s read, which increased <50% of average control amplitude value. For each ASO, the total summed score was calculated and converted to percent of control. Calcium oscillation scores for all ASOs are available in Supplementary Table S1. Representative traces of FLIPR fluorescence signal reads are included in Supplementary Fig. S1.

### Statistical modeling and sequence analysis

The association between sequence features of each ASO and calcium oscillation scores was analyzed by nonparametric Spearman's rank order correlation. Test for significance of this correlation was done using an asymptotic approximation to the Student's *t*-distribution. The calculated features included ASO length, number of LNA-modified sugars, the number of each type of nucleobase, and the length of the stretch of nucleotides from each end of the ASO, which did not include guanine nucleobases. Sequence features analyzed in this study are available in Supplementary Table S1 for all ASOs.

The relation between informative sequence features and calcium oscillation scores was modeled as a weighted linear combination (Eqn. 1). Parameters of the linear model were estimated by the method of least squares and solved numerically by QR decomposition using Householder reflections. This numerical method is implemented in the lm function in the R language for statistical programming [26]. Significance of contributions of each model term to the overall fit was calculated using the *t*-statistic. The trained model is implemented as an R function with source code available in Supplementary Methods.

## Results

### ASOs dosed ICV in mice can be systematically scored for a range of neurobehavioral side effects

We assessed 148 LNA-modified ASOs targeting the premessenger RNA (mRNA) of human microtubule-associated protein Tau (*MAPT*) for behavioral side effects after ICV injection in mice. Five neurobehavioral categories were observed up to 1 h after dosing, and each scored from 0 to 4. Scores in each category were summed to a final acute tolerability score between 0 (no signs observed) to 20 (severe side effects requiring euthanasia).

We chose to evaluate behaviors during the 1st hour for all ASOs because observations of an initial set of 9 ASOs showed that acute behaviors manifested strongly within the 1st hour of dosing and waned anywhere between 2 and 4 h post-dose, depending on the animal and ASO. From 24 h and out to 7 days, we almost never observed any acute behavior reappearing (Supplementary Fig. S2A). Also, for this initial set, mice were injected with doses ranging from 12.5 to 200 μg, and dose-dependent increases in acute tolerability scores were observed for most ASOs (Supplementary Fig. S2A).

For mice dosed at 100 μg, we observed clear separation of ASOs based on their tolerability scores. Furthermore, at this dose, we generally expect robust mRNA knockdown by >50%, as inferred by evaluation of five ASOs in this set by quantitative reverse transcription polymerase chain reaction of samples taken from the brain 7 days after treatment (Supplementary Fig. S2B). Based on these observations, we therefore chose to evaluate all ASOs at a dose of a 100 μg for consistency.

**FIG. 1.**
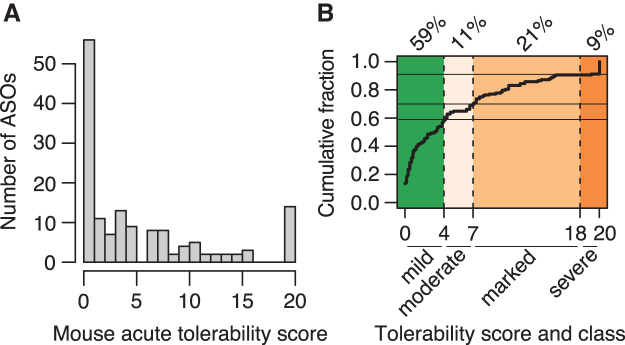
Distribution and grouping of acute tolerability scores. **(A)** Distribution of tolerability scores represented by histogram as *gray bars*. **(B)** Cumulative distribution of tolerability scores with ASOs grouped into mild (scores <4), moderate (scores between 4 and 7), marked (scores between 7 and 18), and severe (scores >18) tolerability classes. Percentages indicate fraction of ASOs in each tolerability class. ASOs, antisense oligonucleotides.

The distribution of observed tolerability scores for the 148 ASOs shows a clear peak close to 0, but almost all scores between 0 and 20 are realized by one or more ASOs (Fig. 1A). Based on inspection of the cumulative distribution of tolerability scores (Fig. 1B), we divided ASO into those with mild, moderate, marked, and severe tolerability signs using score cutoffs at 4, 7, and 18. We judged that only ASOs with no or mild tolerability signs, corresponding to roughly 60% of all ASOs assessed, were suitable for further development. The remaining 40% of all ASOs, with moderate to severe tolerability signs, were judged as having an acute neurotoxic potential too high for further development and evaluation in mouse models.

### Role of NMDA and AMPA receptors in mediating spontaneous calcium oscillations after ASO treatment

Normal function of the CNS depends critically on a wide range of highly regulated processes, including intracellular calcium homeostasis, neurotransmitter release, and electrical activity. The behavioral side effects observed in our studies for some ASOs after dosing to the CNS indicate dysregulation of one or more of these processes. Because of the occurrence of these signs within minutes after dosing, we hypothesized that they were mediated by a rapid onset non-RNA hybridization-based mechanism. Spatial and temporal abnormalities of cytosolic calcium oscillations are known to impact the normal physiological functions of neuronal cells on this timescale [27], and might therefore be implicated.

Spontaneous calcium oscillations have also previously been reported to be affected by neurotoxic small molecules known to cause convulsions [28], and several cellular neurotoxicity assays have been developed based on measurement of intracellular calcium concentrations and dynamics [29]. We therefore next evaluated two ASOs (a toxic ASO resulting in marked tolerability signs and a safe ASO displaying only mild tolerability signs) for effects on spontaneous calcium oscillations, by optical recordings in primary neuronal cells incubated with a fluorescent calcium indicator [25].

**FIG. 2.**
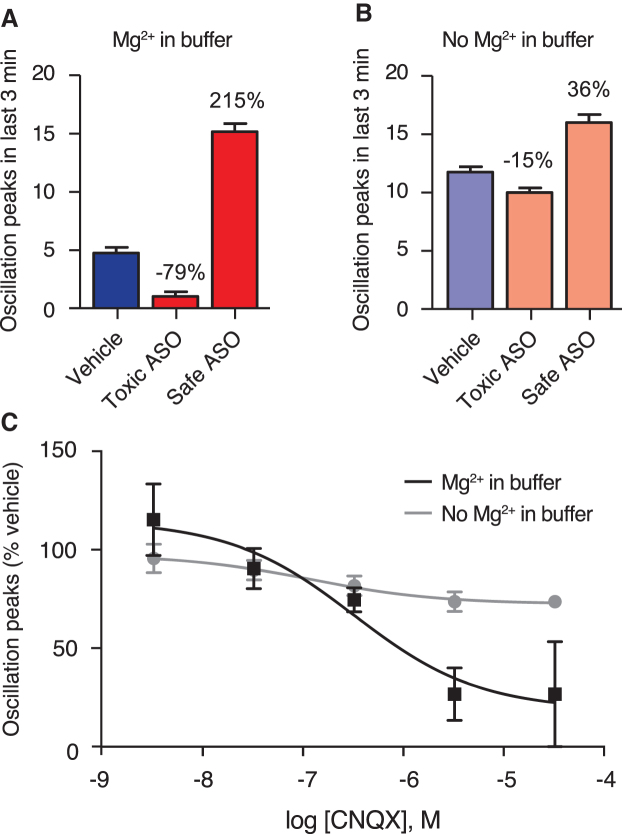
Effect of Mg^2+^ and CNQX on calcium oscillations. The number of oscillations measured over 3 min for vehicle control, an ASO with acute tolerability score of 13 in mice (Toxic ASO), and an ASO with acute tolerability score of 0 (Safe ASO), with **(A)** 1 Mg^2+^ added in the buffer, or **(B)** No Mg^2+^ in the buffer. Percentages indicate increase or decrease relative to vehicle level. *Error bars* indicate 1 SD (*n* = 4). See Supplementary Table S1 for sequence information on ASOs. **(C)** Effect of AMPA receptor antagonist CNQX on number of oscillations measured over 3 min relative to vehicle control in the presence (*black line*) or absence (*gray line*) of Mg^2+^ in the buffer. *Error bars* indicate 1 SD (*n* = 4). Sigmoidal concentration–response curves with variable slopes fitted to data points by *least squares*. AMPA, α-amino-3-hydroxy-5-methyl-4-isoxazolepropionic acid; CNQX, cyanquixaline; SD, standard deviation.

The neuronal calcium oscillation studies were carried out with ASO concentrations at 25 μM, which is within the range of expected concentrations of ASO in mouse CSF the 1st hour after 100 μg ICV injection (data not shown), and with and without 1 mM Mg^2+^ added to the culture media. Compared to vehicle treatment, both in the presence and absence of Mg^2+^, the toxic ASO resulted in fewer calcium oscillations, whereas the safe ASO resulted in more calcium oscillations (Fig. 2A, B). However, we observed five to six times more pronounced effects on oscillations in the presence of Mg^2+^, than when Mg^2+^ was not added, compare Fig. 2A and B.

Both NMDA and AMPA receptors can mediate neuronal calcium oscillations, and Mg^2+^ is known to inhibit NMDA receptors [25]. We therefore speculated that the pronounced changes in calcium oscillations observed for each of the two ASOs in the presence of Mg^2+^ were primarily AMPA dependent. To test this, cells were incubated with AMPA receptor antagonist CNQX at five different concentrations, with or without 1 mM Mg^2+^ in the culture media. In the absence of Mg^2+^, CNQX had minimal inhibitory effects on calcium oscillations (gray points and line in Fig. 2C). However, in the presence of Mg^2+^, CNQX inhibited calcium oscillations down to 25% of vehicle at the highest concentration (black points and line in Fig. 2C).

Slice electrophysiology experiments confirmed that acute addition of toxic ASOs inhibited hippocampal spontaneous excitatory postsynaptic currents (data not shown).

### Acute tolerability correlates with reductions in spontaneous calcium oscillations in neuronal cells

**FIG. 3.**
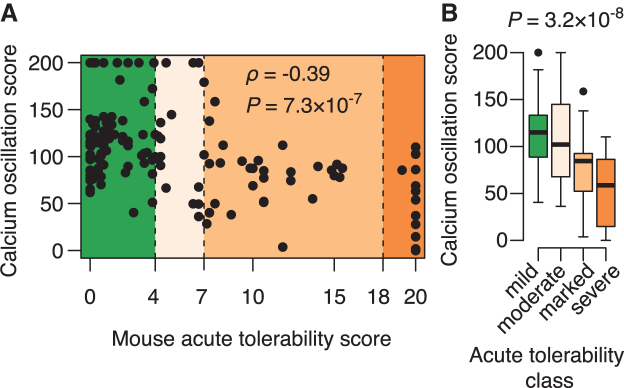
Association between acute tolerability and reductions in spontaneous calcium oscillations in neuronal cells. **(A)** Scatter plot of acute tolerability scores assessed in mice versus calcium oscillation scores evaluated in cells for *n* = 148 ASOs. Nonparametric correlation coefficient calculated as Spearman's rank correlation (⍴*)* with test for significance (*P*) using an asymptotic approximation to the Student's *t*-distribution. **(B)** Box plots showing distributions of calcium oscillation scores for ASOs grouped into mild (scores <4), moderate (scores between 4 and 7), marked (scores between 7 and 18), and severe (scores >18) tolerability classes based on the tolerability scores assessed in mice. Significance (*P*) evaluated by nonparametric Kruskal–Wallis rank sum test.

We next evaluated the 148 ASOs scored for acute tolerability in mice in the calcium oscillation assay (in the presence of Mg^2+^) and found a significant correlation between acute tolerability scores and the calcium oscillation scores evaluated in cells (Fig. 3A). Generally, the more severe the behavioral signs, the larger the reduction in spontaneous calcium oscillations. When grouping the acute tolerability scores into mild, moderate, marked, and severe, the distributions of calcium oscillation scores in each tolerability class were also significantly different (Fig. 3B).

In particular, for the marked and severe tolerability classes with tolerability scores above 7, the calcium oscillations scores were almost always found to be reduced below pretreatment levels. Taken together, the data presented in Figs. 2 and 3 support a link between acute tolerability observed *in vivo* and *in vitro* effects on AMPA-dependent calcium oscillations. We have not observed correlations between acute tolerability scores in mice and any other type of cellular toxicity responses such as induction of caspase or inhibition of tubulin (data not shown).

### Associations between sequence features and calcium oscillation scores

**FIG. 4.**
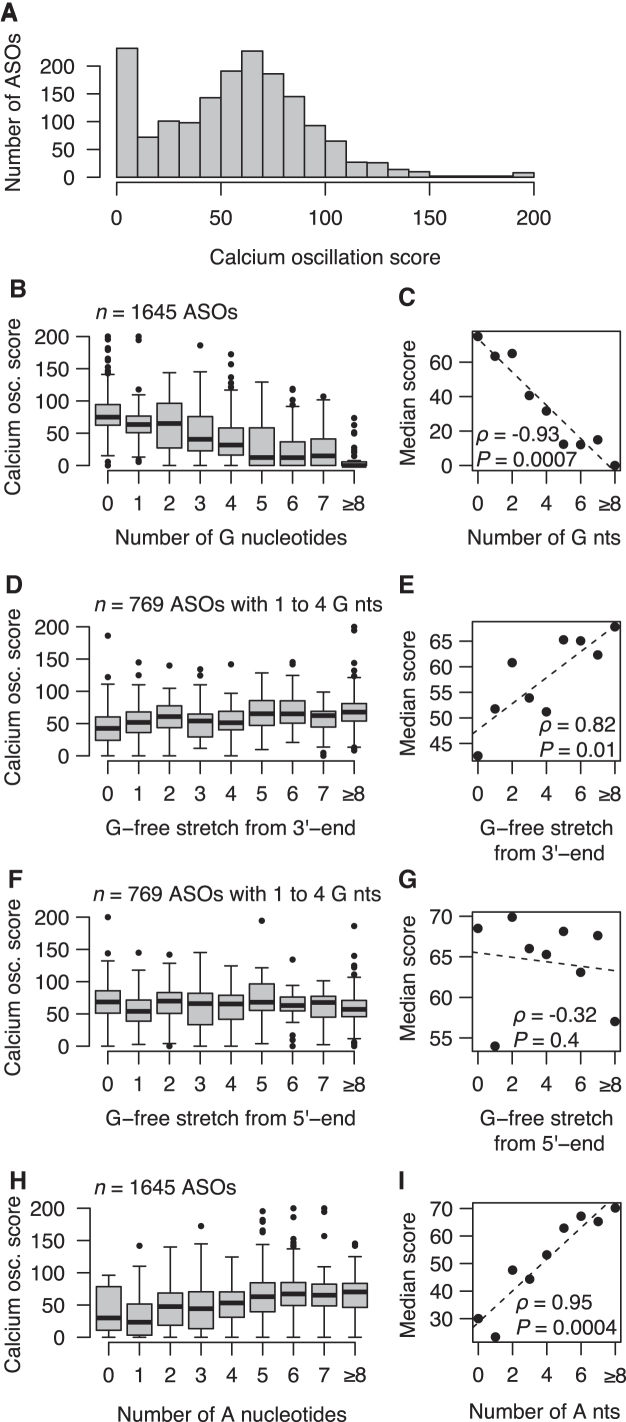
Associations between sequence features and calcium oscillation scores. **(A)** Distribution of calcium oscillation scores evaluated in neuronal cells represented by histogram as *gray bars* for *n* = 1,645 ASOs. Scores >200 were set to 200. **(B)** Box plots showing distributions of calcium oscillation scores for ASOs grouped based on the number of guanine (G) nucleotides. **(C)** Scatter plot of median calcium oscillation scores for ASOs with a given number of G nucleotides. Nonparametric correlation coefficient calculated as Spearman's rank correlation (⍴) with test for significance (*P*) using an asymptotic approximation of the Student's *t*-distribution. *Dashed trend line* calculated by linear least-square fitting. **(D)** Distributions of calcium oscillation scores for ASOs with 1 to 4 G nucleotides, grouped based on the length of the stretch of nucleotides counting from the 3′ end that are not G nucleotides. **(E)** Similar to **(C),** but for ASOs with 1 to 4 G nucleotides grouped based on the length of the G-free stretch from the 3′ end. **(F, G)** Similar to **(D)** and **(E)**, but with ASOs grouped based on the length of the stretch of nucleotides counting from the 5′-end. **(H, I)** Similar to **(B)** and **(C)**, but for adenosine **(A)** nucleotides.

Because of the translatability between the calcium oscillation scores evaluated in cells and the acute behavioral scores assessed in mice (Fig. 3), we evaluated in cells an additional 1,645 ASOs targeting the *MAPT* pre-mRNA. These ASOs had not been selected for dosing in mice because of weaker on-target activity when evaluated in cells (data not shown). We observed a broad range of AMPA-dependent calcium oscillation scores in this set of ASOs, indicating a highly variable association between the ASO sequence and its effect on spontaneous calcium oscillations (Fig. 4A). We observed that similar sequences (as calculated by the Levenshtein edit distance) generally tend toward having more similar calcium oscillation scores than less similar sequences (Supplementary Fig. S3A). This trend was also observed for mouse tolerability scores (Supplementary Fig. S3B).

The 1,645 ASOs evaluated were mostly between 14 and 20 nt in length, and with between 3 and 9 LNA-modified nucleotides in total in the flanks. However, neither length nor the number of LNA modifications could directly explain the calcium oscillation scores (Supplementary Fig. S4). It follows from this that gap length (the number of consecutive DNA nucleotides in the middle region of the ASO) does not explain the calcium oscillation scores either, since gap length is essentially the same as the difference between overall length and number of LNA modifications in the flanks.

We therefore next turned to analyzing the base sequence composition of ASOs in relation to the calcium oscillation scores. Since the ASOs were originally designed to comprehensively cover the *MAPT* pre-mRNA, to best allow regions supporting effective knockdown to be identified, the base compositions were highly variable between ASOs. Specifically, the training set of 1,645 ASOs is targeted to 313 different and nonoverlapping regions on the *MAPT* pre-mRNA and in total cover 7,063 nts (Supplementary Fig. S5A).

As seen in Fig. 4B, C, we found that for the guanine nucleobase (G), the more G nucleotides in the ASOs, the lower the calcium oscillation scores. Furthermore, the shorter the stretch of nucleotides from the 3′-end of the ASO, but not the 5′-end, which did not include G, the lower the calcium oscillation scores (Fig. 4D–G). Oppositely, for the adenine nucleobase (A), the more A nucleotides in the ASOs, the higher the calcium oscillation scores (Fig. 4H, I). For the number of thymine (T) and cytosine (C) nucleotides, their direct associations to calcium oscillation scores were also positively correlated with calcium oscillation scores, most strongly for C nucleotides, although for neither of them was the correlation as strong as for the A nucleotides (Supplementary Fig. S6).

### Linear combination of informative sequence features accurately predicts acute neurotoxic potential

**FIG. 5.**
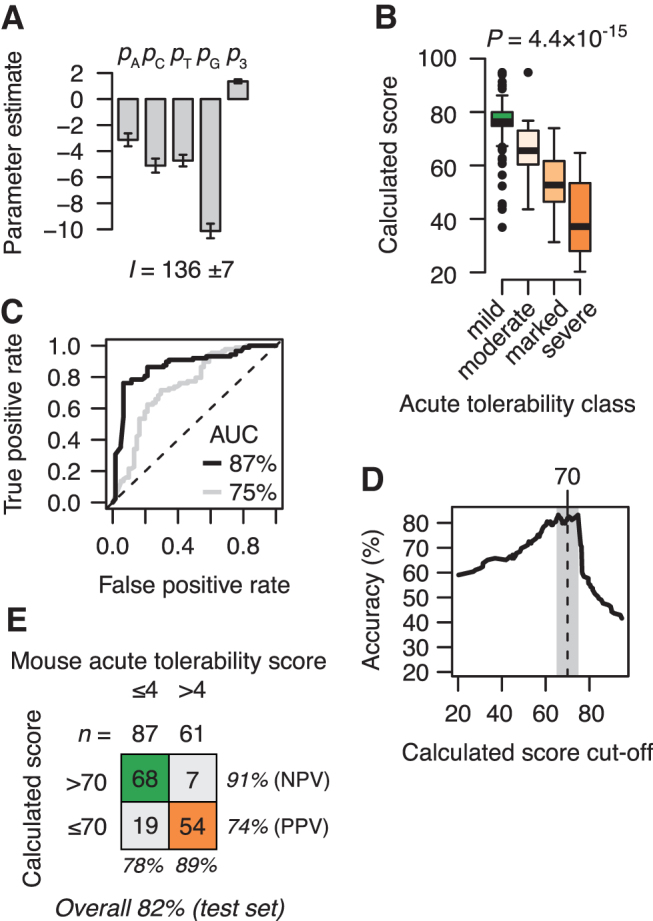
Linear combination of informative sequence used to classify acute tolerability classes in mice for a test set of ASOs. **(A)** Fitted parameters for the linear model (Equation 1). Error bars indicate 1 SD. **(B)** Box plots showing distributions of calculated scores for ASOs in the test set of *n* = 148 ASOs grouped into mild, moderate, marked, and severe tolerability classes based on the tolerability scores assessed in mice. Significance (*P*) evaluated by nonparametric Kruskal–Wallis rank sum test. **(C)** Receiver operating characteristics curve for acute tolerability scores grouped into ASOs with mild (scores ≤4) or moderate to severe (scores >4) tolerability scores, varying the score threshold calculated from the linear model (*black line*), or the measured calcium oscillations (*gray line*). **(D)** Accuracy of the classification of ASOs in the test set as a function of the cutoff chosen for the scores calculated from the linear model. *Gray area* indicates the score region from 65 to 75 where highest overall accuracy is observed. *Vertical dashed line* indicates midpoint of this score range. **(E)** Classification performance in the test set using scores calculated from the linear model with optimal cutoff score at 70. AUC, area under the curve; NPV, negative predictive value; PPV, positive predictive value.

Inspection of the association between informative sequence features and median calcium oscillation scores in Fig. 4 and Supplementary Fig. S6 reveals an approximately linear relation. We therefore summarized these features as a weighted linear combination, see Equation 1 below

(1) $$score=p_{A}n_{A}+p_{T}n_{T}+p_{C}n_{C}+p_{G}n_{G}+p_{3}g_{3}+I$$

where *n*_A_, *n*_T_, *n*_C_, and *n*_G_ are the numbers of each type of nucleotide in the ASO sequence, *g*_3_ is the length of the G-free stretch from the 3′-end, and *p*_A_, *p*_T_, *p*_C_, *p*_G_, and *p*_3_ are the corresponding weighted parameter values. These parameters, as well as the intercept *I*, were determined by least-squares fitting of the model to the observed calcium oscillation scores for the 1,645 ASOs not evaluated in mice (in this study, termed the training set) (Fig. 5A). All terms in the model were found to contribute highly significantly to the overall fit (*P* < 4 × 10^–10^ for each parameter based on the *t*-statistic).

The trained linear model reproduces the observation that similar sequences in general have similar scores (Supplementary Fig. S3C). Expansion of the model by inclusion of more complicated ASO features such as dinucleotide or trinucleotide counts did not substantially improve model fit (data not shown) and carries the risk of overfitting the model because of the increasing number of features. Such expanded models were therefore not considered further.

We used the model to calculate scores for the 148 ASOs tested in mice that were set aside and not used to train the model (in this study, termed the test set). We observed a significant correlation with the calcium oscillation scores measured for this set of ASOs (Supplementary Fig. S7A).

Interestingly, the calculated scores correlated even more significantly with the acute tolerability scores observed in mice (Supplementary Fig. S7B). Similarly, inspection of distributions of calculated scores when grouping the ASOs into the four tolerability classes (Fig. 5B), again revealed a more significant separation of ASOs compared to what the measured calcium oscillation scores achieved, compare Figs. 3B and 5B. These results suggest that the calculated scores can achieve a higher predictive performance of classifying the acute tolerability behavior observed in mice, compared to the measured calcium oscillation scores.

Supporting this, a receiver operating characteristic curve analysis of classification performance of the calculated score showed an area under the curve (AUC) at 87% (Fig. 5C, black line), whereas for the measured scores, the AUC was at 75% (Fig. 5C, gray line). We speculate that the increased classification performance of the calculated score compared to the measured score can partly be explained by the model being able to capture the underlying effect on calcium oscillations without the biological noise of the measurement procedure. In this sense, the calculated score represents a model-guided average estimate of this observable compared to the measured score. Highest overall accuracy of classification was found at a cutoff threshold for the calculated score at 70 (Fig. 5D). Applying this cutoff resulted in a classification accuracy of 82% (Fig. 5E).

The specificity, or negative predictive value, of the classification is at 91% in the test set. That is, if only ASOs with calculated scores above 70 had been assessed in mice, 91% of these ASOs would have acceptable acute neurotoxic potentials in mice with tolerability scores at or below 4.

### Independent validation of the prediction model

The test set of 148 ASOs targets 44 different and nonoverlapping regions on the MAPT pre-mRNA and in total cover 882 nts (Supplementary Fig. S5B). Although none of the ASOs in the test set was included in the training set, they are targeting regions that are mostly also targeted by ASOs in the test set. Indeed, 813 out of the 882 nts covered (92%) are also among the 7,063 nts covered by the training set (Supplementary Fig. S5A, B). Potential region-specific influences could therefore result in sequence motifs shared by ASOs in both training and test sets and bias the estimate of predictive accuracy from the test set (Fig. 5E).

**FIG. 6.**
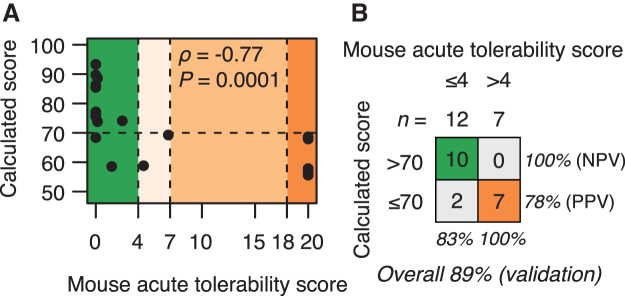
Linear combination of informative sequence used to classify acute tolerability classes in mice for a validation set of ASOs. **(A)** Scatter plot of acute tolerability scores assessed in mice versus scores calculated from the linear model for a validation set of *n* = 19 ASOs. **(B)** Classification performance in the validation set using scores calculated from the linear model with cutoff score at 70.

However, when excluding the ASOs from the training set that overlaps with the regions covered by ASOs from the test set, and re-training the model on this reduced training set, the classification performance remains exactly the same. To further establish this, we next validated the model by calculating scores for ASOs designed to target a different human pre-mRNA. Specifically, we calculated scores for 19 ASOs designed to target the human stanniocalcin 1 pre-mRNA (STC1), which we had also assessed in mice for behavioral side effects. The 19 ASOs target 7 unique and nonoverlapping regions on the STC1 pre-mRNA (Supplementary Fig. S5C). In this validation set of ASOs, 12 had no or only mild tolerability signs with acute behavioral scores below 4, and seven had scores above 4, some even reaching the maximal score of 20 (Fig. 6A).

For this set of ASOs, the calculated scores correlated significantly with the acute behavioral scores, and at a cutoff at 70, achieved an overall classification accuracy of 89% (Fig. 6B). We also measured calcium oscillation scores in neuronal cells for this validation set of ASOs. Using a cutoff at 75 for the measured score, which gave the highest classification accuracy in the training set (Supplementary Fig. S8A), resulted in a slightly less accurate classification accuracy at 84% (Supplementary Fig. S8B).

### Identification of negative control designs with very low acute neurotoxic potential

The analysis of sequence features (Fig. 4) as well as the model parameters estimated from the data (Fig. 5A) highlights the significance of the number and position of G nucleotides toward the 3′-end in the ASOs as increasing the acute neurotoxic potential. In addition, the presence of A nucleotides decreases the acute neurotoxic potential to a larger extent than C and T nucleotides. Consequently, ASOs with no G nucleotide, and a higher proportion of A than C and T nucleotides, should be much more likely to have a low acute neurotoxic potential.

To further test the generality of this observation, we designed 13 ASOs with no G nucleotide (Table 1). For these ASOs, we furthermore required that the base sequence was not fully complementary to any RNA sequence in mice, and that <0.1% of all mouse RNA sequences harbored partially complementary (less than three mismatched base pairs) potential binding regions to any of these ASOs. With this requirement, the ASOs designed could serve as general negative controls for target knockdown in future mouse studies with ICV dosing.

**Table 1.** Negative Control Antisense Oligonucleotides with Low Acute Neurotoxic Potential in Mice

Sequence	*n*_A_	*n*_T_	*n*_C_	*n*_G_	Calculated score	Tolerance score
**TCC**actaaccaatat**AAC**	8	4	6	0	89	0.2
**TAA**ctaacttaactc**AAC**	8	5	5	0	89	0.2
**CAC**aatctaactatt**AAC**	8	5	5	0	89	0.5
**AAA**tctataataaccac**CAC**	10	4	6	0	82	0
**CAC**aactattaaatacc**AAC**	10	4	6	0	82	0.7
**TAC**catacaataacttt**AAC**	9	6	5	0	81	0
**CTA**atattataaccatc**AAC**	9	6	5	0	81	0.2
**TAC**tcaaatataacacc**AAC**	10	4	6	0	82	0.2
**CAAC**caacaatacttt**AAAC**	10	4	6	0	82	0.2
**CAAA**tcatccatctat**AAAC**	9	5	6	0	81	0
**CAAA**cttatatctttc**AAAC**	8	7	5	0	80	0
**CTAA**atccttaatatc**AAAC**	9	6	5	0	81	0
**C**c**AAA**tcttataata**AC**t**AC**	9	6	5	0	81	0

Sequences are shown with LNA-modified nucleotides in upper case bold and DNA nucleotides in lower case. All ASOs are with full phosphorothioate backbones. The ASOs have no perfectly matching or partially mismatched targets in the mouse transcriptome.

ASOs, antisense oligonucleotides; LNA, locked nucleic acid.

Among the random base sequences fulfilling the abovementioned criteria, preference was given to those where the proportion of A nucleotides roughly equaled the sum of T and C nucleotides. Finally, designs with slightly different lengths and LNA modification patterns were chosen. As expected, the calculated scores for these 13 ASOs were all >70, and, when evaluated in mice by ICV dosing, were all found to be well tolerated with acute tolerability scores below 1 (Table 1).

## Discussion

We have, in this study, reported on the serious neurobehavioral side effects we observed for two out of every five LNA-modified PS-ASOs injected into cerebral lateral ventricles in mouse brain. Across hundreds of ASOs evaluated in this manner, for those ASOs where the adverse effects manifested, they typically occurred within minutes after dosing. We have found that ASOs with high potential for this type of acute neurotoxicity often cause reductions in spontaneous calcium oscillations in primary neuronal cells, and that their nucleobase composition differs significantly from ASOs with lower acute neurotoxic potential.

We expect these results to apply to ASOs irrespective of their intended function, be that directing enzymes to act on the RNA target, blocking motifs in the RNA sequence, or affecting the RNA structure. These insights allow the acute neurotoxic potential of LNA-modified PS ASOs to be managed both *in silico* and *in vitro* before ICV dosing in mice, resulting in high impact on drug discovery campaigns: (1) the identification of safe tool compounds needed to test disease hypotheses *in vivo*, (2) a significant reduction in animals needed for ASO testing and improvement in animal welfare, and (3) reduced risk of testing potentially toxic ASOs in higher species.

We speculate that additional factors beside the impact on spontaneous calcium oscillations may also contribute to the acute neurotoxic potential observed in mice. In particular, for calcium oscillation scores ∼100 (Fig. 3A), in mice, these ASOs are spread across all tolerability classes. A counter screen in another cellular assay might help explain these differences in mice, and thereby help improve overall predictive accuracy in cells as well as provide possibilities for further development of the computational model.

A total of 1,825 ASOs were evaluated in cells and mice in this study, all with LNA-modified flanks and a central DNA region supporting RNase H binding. We did not see any difference between sugar-modified (LNA) nucleotides and DNA nucleotides with respect to their contribution to the acute neurotoxic potential, suggesting that they impact this potential to the same extent. However, future work, for example with fully sugar-modified oligonucleotides as well as with oligonucleotides with no sugar modifications, should explore this further.

Future work should also explore a wider range of sugar modifications to establish whether some sugar modifications impact the frequency and severity of these side effects to a different extent than others. Acute toxicity of ASOs with deoxy-, cET-, and 2′-O-methoxyethyl modifications to the sugar ring has been reported in patent publications in both mice and rats regardless of route of administration (intrathecal vs. ICV) [22–24]. ASOs in these publications as well as ASOs reported in this study could provide starting points for such future work.

We only evaluated ASOs with full PS backbones in these studies. Future work should consider the extent to which this backbone modification chemistry contributes to the acute neurotoxic potential compared to other types of backbone chemistries. Similarly, ASOs where ligands have been conjugated at one or both ends might present a different profile compared to the unconjugated ASOs evaluated in this study.

Comparing the informative features identified in this work for predicting acute neurotoxic potential with earlier work identifying features for predicting hepatotoxic potentials [17], the model presented in this work generally relies on fewer and simpler features. In both cases, ASOs are represented as strings of characters and features are derived from that. However, for predicting acute neurotoxic potential, a linear combination of counts and positions of single nucleotides is sufficient, whereas for predicting hepatotoxic potential, an ensemble of decision trees trained on a much larger feature set of dinucleotide counts that take sugar modifications into account was needed.

When evaluating decision tree ensemble models similar to what was reported for hepatotoxic potentials [17], but trained on the calcium oscillation data, we did not observe improved predictive accuracy (data not shown). We speculate that a reason why the linear model presented in this work is sufficient, could simply be that the mechanisms that can lead to toxicity are different for acute neurotoxicity and hepatotoxicity. This is supported by the rapid onset of acute neurotoxicity within minutes after injection compared to hepatotoxicity that typically manifests days to weeks after injection.

The underlying mechanism explaining why G nucleotides increase the acute neurotoxic potential the most, and A nucleotides the least, is unclear to us. We speculate that it could be connected to basic chemical properties of the four different nucleotides. For example, guanine has the lowest redox potential of the four nucleobases, and is therefore the most easily oxidized [30,31]. It could also be that the ASOs directly or indirectly act as calcium chelators, which might explain the effects on spontaneous calcium oscillations.

In support of this, it has previously been described that oligonucleotides can inhibit oxidation reactions primarily by a mechanism of metal ion chelation [32]. The negative charge of the ASOs tested could also be considered affecting the ion flux and leading to neuronal hyperexcitability and eventually seizures [33].

Our data also suggest a link between AMPA receptors, ASO sequence, and acute *in vivo* tolerability. While the mechanism of this remains to be determined, one possibility is that ASOs, when taken up into neurons in mouse brain, transiently affect the membrane trafficking of AMPA receptors. This could be either by direct interactions, or indirectly, perhaps mediated by some of the chemical properties discussed above. This disruption of AMPA receptor trafficking and normal calcium transients could dynamically disrupt neuronal network activity, leading to an imbalance between excitatory and inhibitory neuronal firing in the mouse brain. Future work could explore if the findings reported in this study directly translate into changes in neuronal activity such as depression of the CNS.

These or other chemical properties potentially impacting the acute neurotoxic potential are likely affected by whether nucleobases are paired as double-stranded oligonucleotides or not. We consequently speculate that small interfering RNAs [34,35] or ASOs duplexed with complementary RNA [36] could present a different profile after ICV administration compared to the single-stranded ASOs evaluated in this study.

Furthermore, it may be possible to “dampen” the chemical properties affecting the acute neurotoxic potential, either by formulating ASOs in complex solutions instead of the simple saline solution used in these studies or by chemically modifying the guanine without impacting its binding properties negatively [37]. This last approach has similarities to what has been suggested for reducing the immunostimulatory potential associated with CG dinucleotides in some ASOs [38]. These considerations could suggest areas in which to focus future medicinal chemistry efforts.

## Supplementary Material

Supplemental data

Supplemental data

Supplemental data

Supplemental data

Supplemental data

Supplemental data

Supplemental data

Supplemental data

Supplemental data

Supplemental data
