# Supplementary material for: Acute Neurotoxicity of Antisense Oligonucleotides After Intracerebroventricular Injection Into Mouse Brain Can Be Predicted from Sequence Features
Source: Nucleic Acid Ther. 2022 Jun 1;32(3):151–62. doi: 10.1089/nat.2021.0071 (PMC9221153; doi:10.1089/nat.2021.0071)
Supplement: Supplemental data [file Suppl_FigureS1.docx]

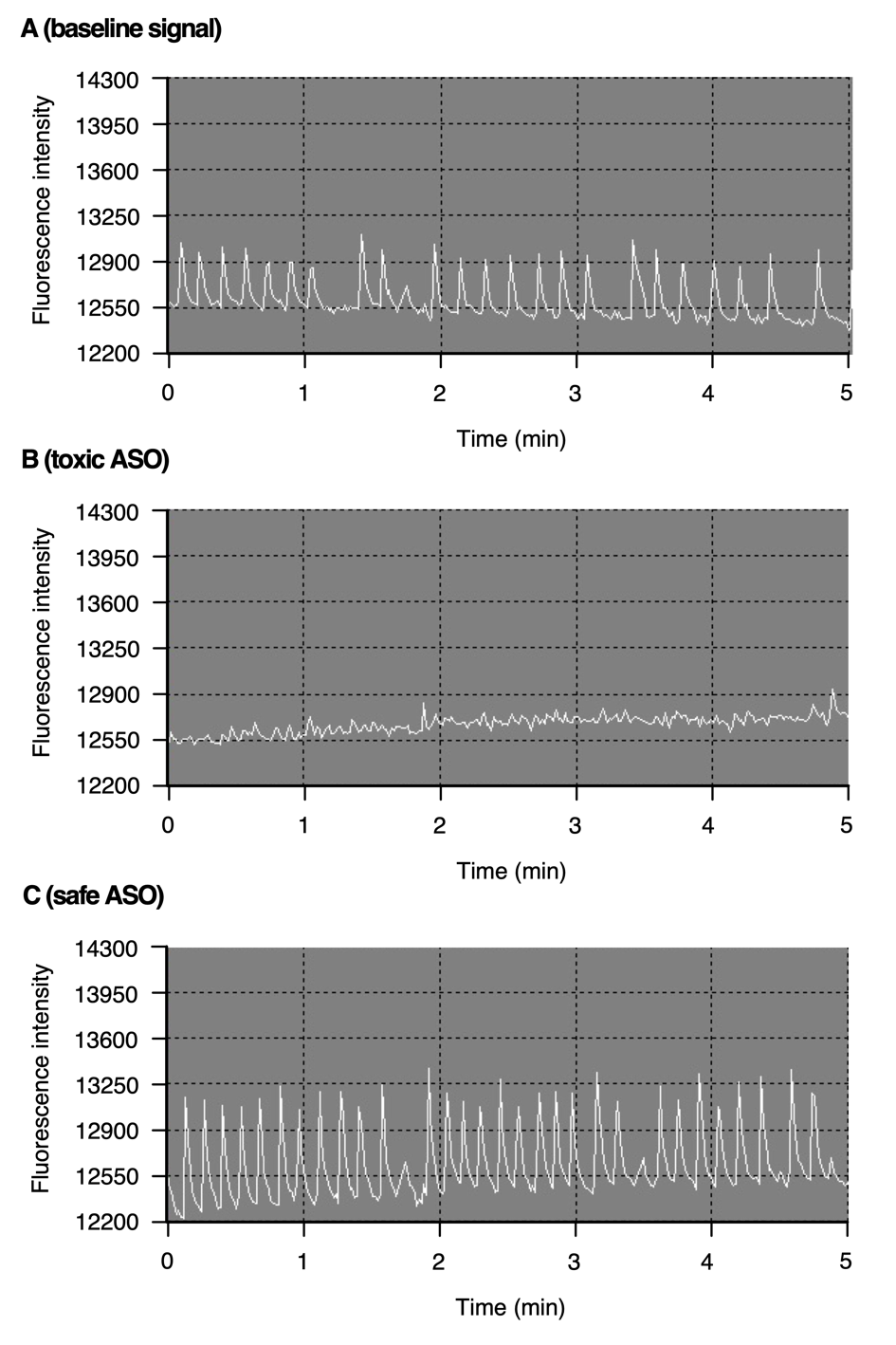


**Figure S1** *Representative calcium oscillations in cortical neurons measured by FLIPR assay as traces of fluorescence levels in a 5-minute time window* **A)** Baseline signal of intracellular calcium oscillations in the absence of ASO. The average control amplitude value for the 24 peaks detected is 584 units. **B)** Calcium oscillations for a toxic ASO, see Suppl. Table 1. Only two peaks (circled) have amplitudes > 292 units (50% of average control amplitude). As percent of control the calcium oscillation score is 2/24 * 100 = 8. **C)** Calcium oscillations for a safe ASO, see Suppl. Table 1. In all 31 peaks have amplitudes > 292 units. The calcium oscillation score is 31/24 *100 = 129.
