# Supplementary material for: Acute Neurotoxicity of Antisense Oligonucleotides After Intracerebroventricular Injection Into Mouse Brain Can Be Predicted from Sequence Features
Source: Nucleic Acid Ther. 2022 Jun 1;32(3):151–62. doi: 10.1089/nat.2021.0071 (PMC9221153; doi:10.1089/nat.2021.0071)
Supplement: Supplemental data [file Suppl_Data.docx]

**SUPPLEMENTARY METHODS**

**Computer code for calculating acute neurotoxicity scores**

The trained model is implemented as a function in the R programming language as shown below

calculate_acute_neurotox <- function(xx) {

*## make sure input is in character format* xx <- as.character(xx)

 *## count the number of each nucleotide*
 lf <- function(x) {
 x <- tolower(x)
 x <- strsplit(x, "")[[1]]
 x <- table(factor(x,levels=c("a","c","t","g")))
 return(x)
 }
 cnt_nt <- as.data.frame(t(sapply(xx, lf)))

*## count number of nucleotides from the 3'-end that are not g*
 gfree3 <- function(x) {
 x <- tolower(x)
 x <- strsplit(x, "")[[1]]
 tfg <- x=="g"
 if (sum(tfg)==0) {
 l3 <- NA
 } else {
 posg <- c(1:length(x))[tfg]
 l3 <- length(x)-max(posg)
 }
 return(l3)
 }
 cnt_gfree3 <- sapply(xx, gfree3)
 cnt_gfree3[cnt_gfree3>20] <- 20 *#Set max to 20*
 cnt_gfree3[is.na(cnt_gfree3)] <- 20 *#Set no g in ASO to 20*

*## Calculate final score based on trained parameters and return result* calc_out <- round(136.0430 - 3.1263*cnt_nt$a - 5.1100*cnt_nt$c -
 4.7217*cnt_nt$t - 10.1264*cnt_nt$g + 1.3577*cnt_gfree3,1)

 return(as.numeric(calc_out))

}

To load the function, copy/paste the code (text in Courier font) to the R programming environment.

**Example**

Once the calculate_acute_neurotox function has been loaded into the R programming environment, scores can be calculated for ASO sequences. As an example, scores are calculated for the sequences AAAtctataataaccacCAC, CAAAtcatccatctatAAAC, and TACcatacaataactttAAC and stored in the vector calc_out using the following command in R

calc_out <- calculate_acute_neurotox(c("AAAtctataataaccacCAC", "CAAAtcatccatctatAAAC", "TACcatacaataactttAAC"))
