# Supplementary material for: Acute Neurotoxicity of Antisense Oligonucleotides After Intracerebroventricular Injection Into Mouse Brain Can Be Predicted from Sequence Features
Source: Nucleic Acid Ther. 2022 Jun 1;32(3):151–62. doi: 10.1089/nat.2021.0071 (PMC9221153; doi:10.1089/nat.2021.0071)
Supplement: Supplemental data [file Suppl_FigureS2.docx]

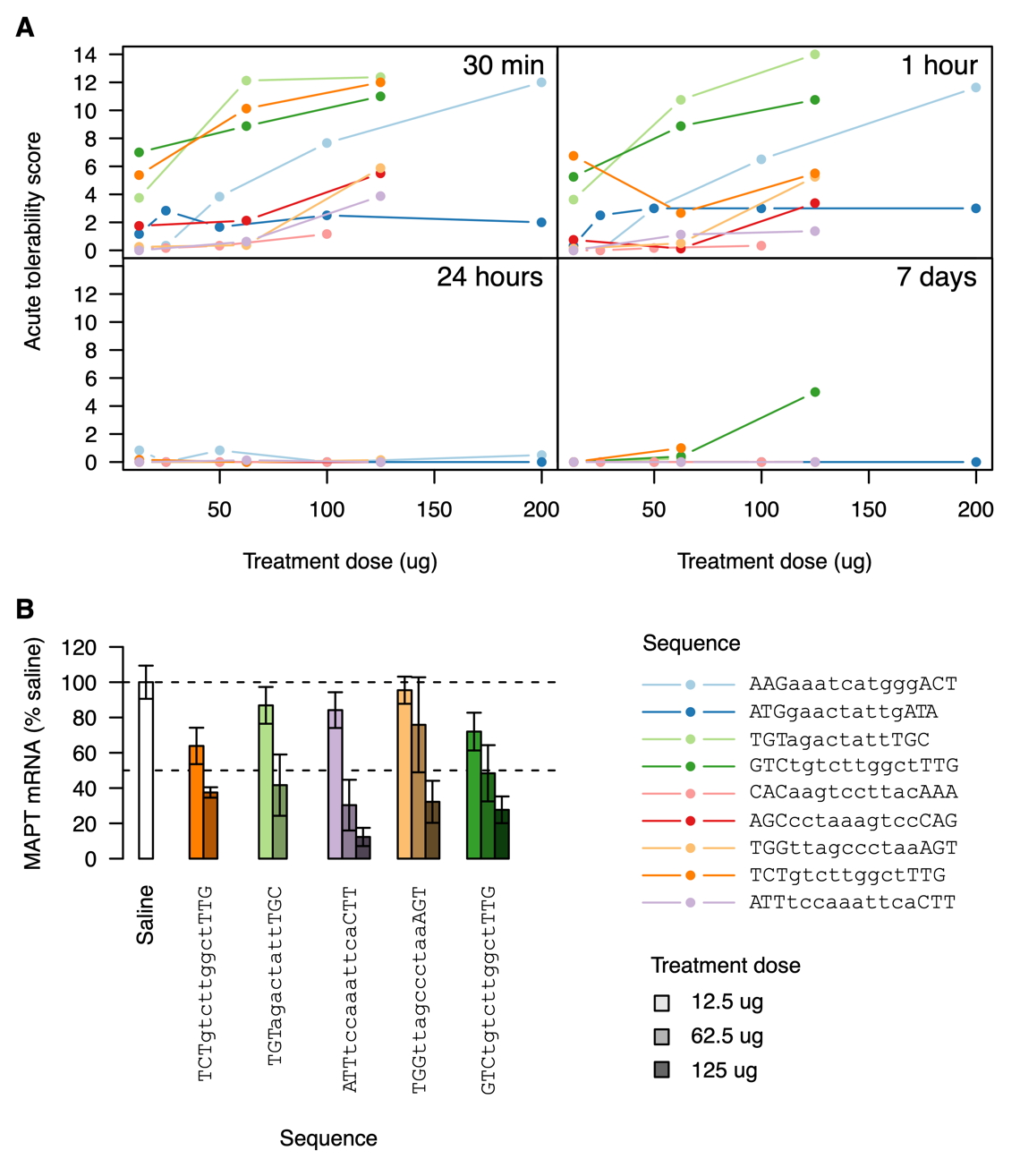


**Figure S2** *Dose response evaluations of ASOs* **A)** Evaluation of acute tolerability scores for 9 ASOs at 30 min, 1 hour, 24 hours, and 7 days after injection of 12.5, 25, 50, 62.5, 100, 125 and 200 ug (depending on the ASO). **B)** Evaluation of MAPT mRNA knockdown in brain samples 7 days after treatment with 5 out of the 9 ASOs at three different doses. Error bars indicate 1 SD (*n* = 8).
