# Supplementary material for: Acute Neurotoxicity of Antisense Oligonucleotides After Intracerebroventricular Injection Into Mouse Brain Can Be Predicted from Sequence Features
Source: Nucleic Acid Ther. 2022 Jun 1;32(3):151–62. doi: 10.1089/nat.2021.0071 (PMC9221153; doi:10.1089/nat.2021.0071)
Supplement: Supplemental data [file Suppl_FigureS3.docx]

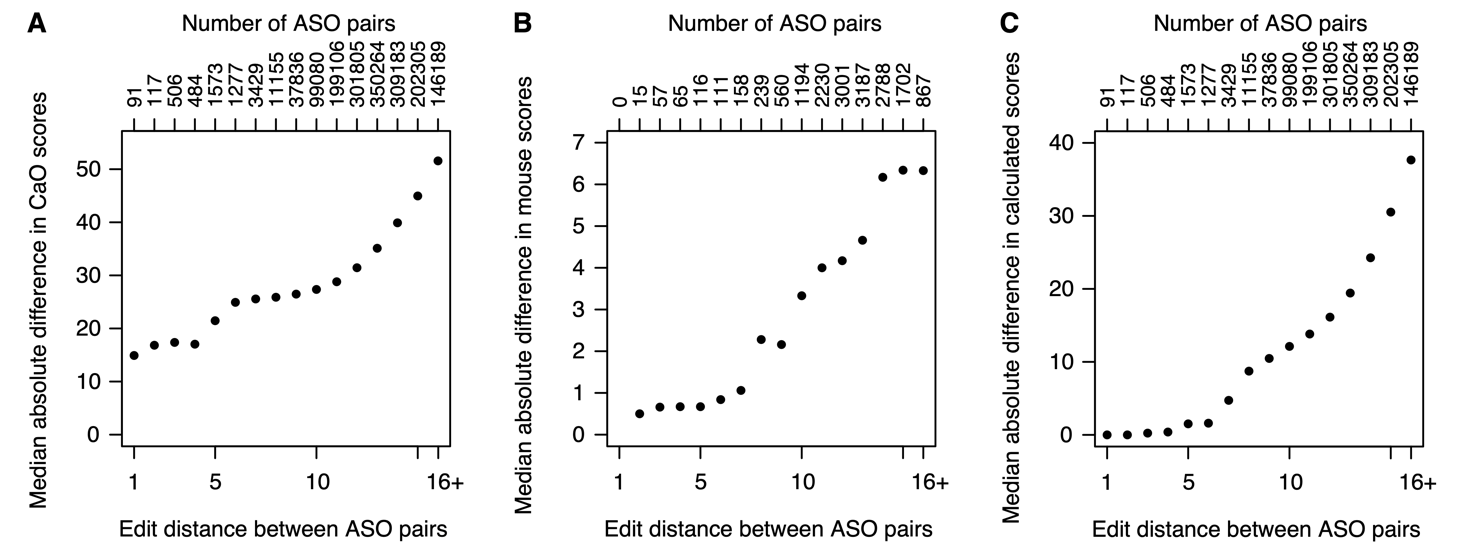


**Figure S3** *Similar sequences generally have more similar toxicity scores* **A)** For all possible pairs of ASOs with calcium oscillation scores, the Levenshtein edit distance between the ASO sequences and the mean absolute difference in calcium oscillation scores were calculated. Pairs were grouped by edit distance (*x*-axis) and the median of the absolute differences calculated for each group (*y*-axis). **B)** Same analysis for mouse tolerability scores. **C)** Same analysis for scores calculated by the model (Eqn. 1).
