# Supplementary material for: Acute Neurotoxicity of Antisense Oligonucleotides After Intracerebroventricular Injection Into Mouse Brain Can Be Predicted from Sequence Features
Source: Nucleic Acid Ther. 2022 Jun 1;32(3):151–62. doi: 10.1089/nat.2021.0071 (PMC9221153; doi:10.1089/nat.2021.0071)
Supplement: Supplemental data [file Suppl_FigureS4.docx]

| 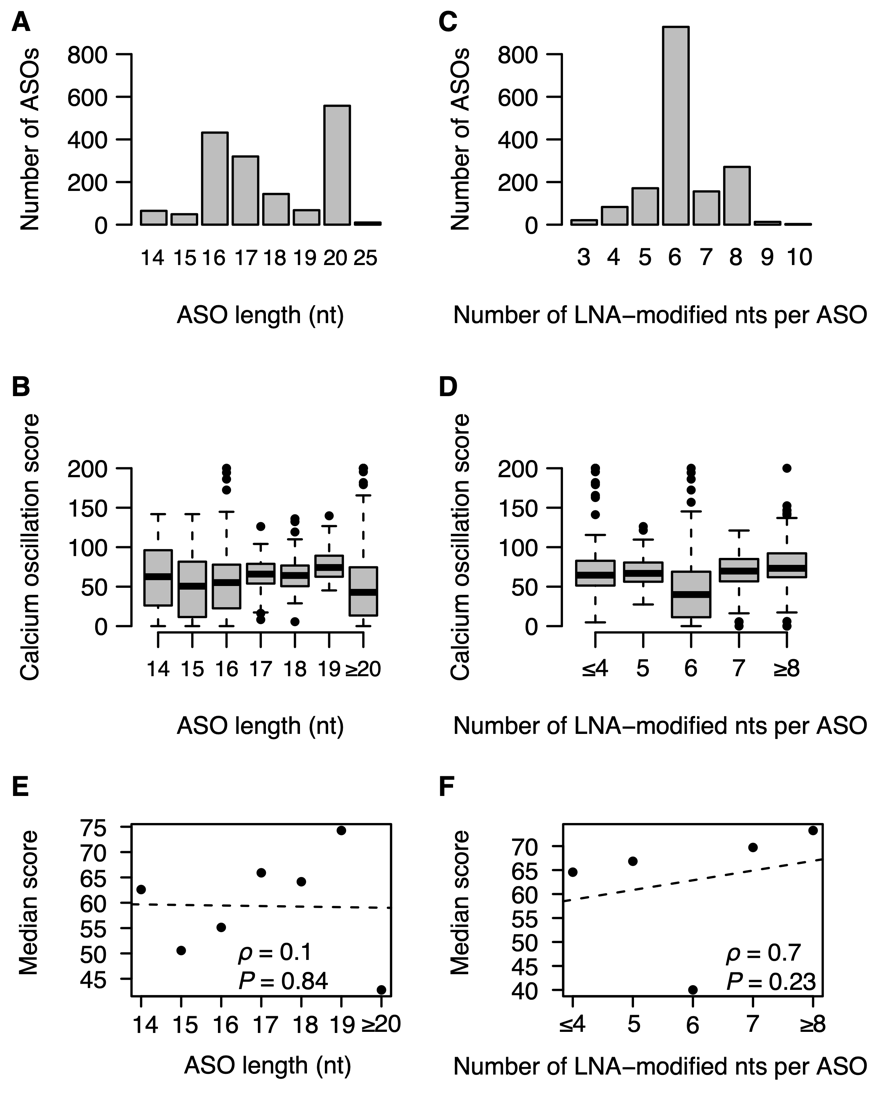 |
| --- |
| **Figure S4** *Associations between additional sequence features and calcium oscillation scores* **A)** Distribution of ASO lengths for all ASOs evaluated in neuronal cells for calcium oscillations. **B)** Boxplots of calcium oscillation scores stratified by ASO length. **C)** Distribution of the number of LNA-modified nucleotides per ASO for all ASOs. **D)** Boxplots of calcium oscillation scores stratified by number of LNA-nucleotides per ASO. **E)** Scatterplot of median calcium oscillation scores stratified by ASO length. Nonparametric correlation coefficient calculated as Spearman's rank correlation (*⍴)* with test for significance (*P*) using an ﻿asymptotic approximation of the Student's *t*-distribution. Dashed trend line calculated by linear least-square fitting. **F)** Same as E), but stratified by number of LNA-nucleotides. |
