# Supplementary material for: Acute Neurotoxicity of Antisense Oligonucleotides After Intracerebroventricular Injection Into Mouse Brain Can Be Predicted from Sequence Features
Source: Nucleic Acid Ther. 2022 Jun 1;32(3):151–62. doi: 10.1089/nat.2021.0071 (PMC9221153; doi:10.1089/nat.2021.0071)
Supplement: Supplemental data [file Suppl_FigureS5.docx]

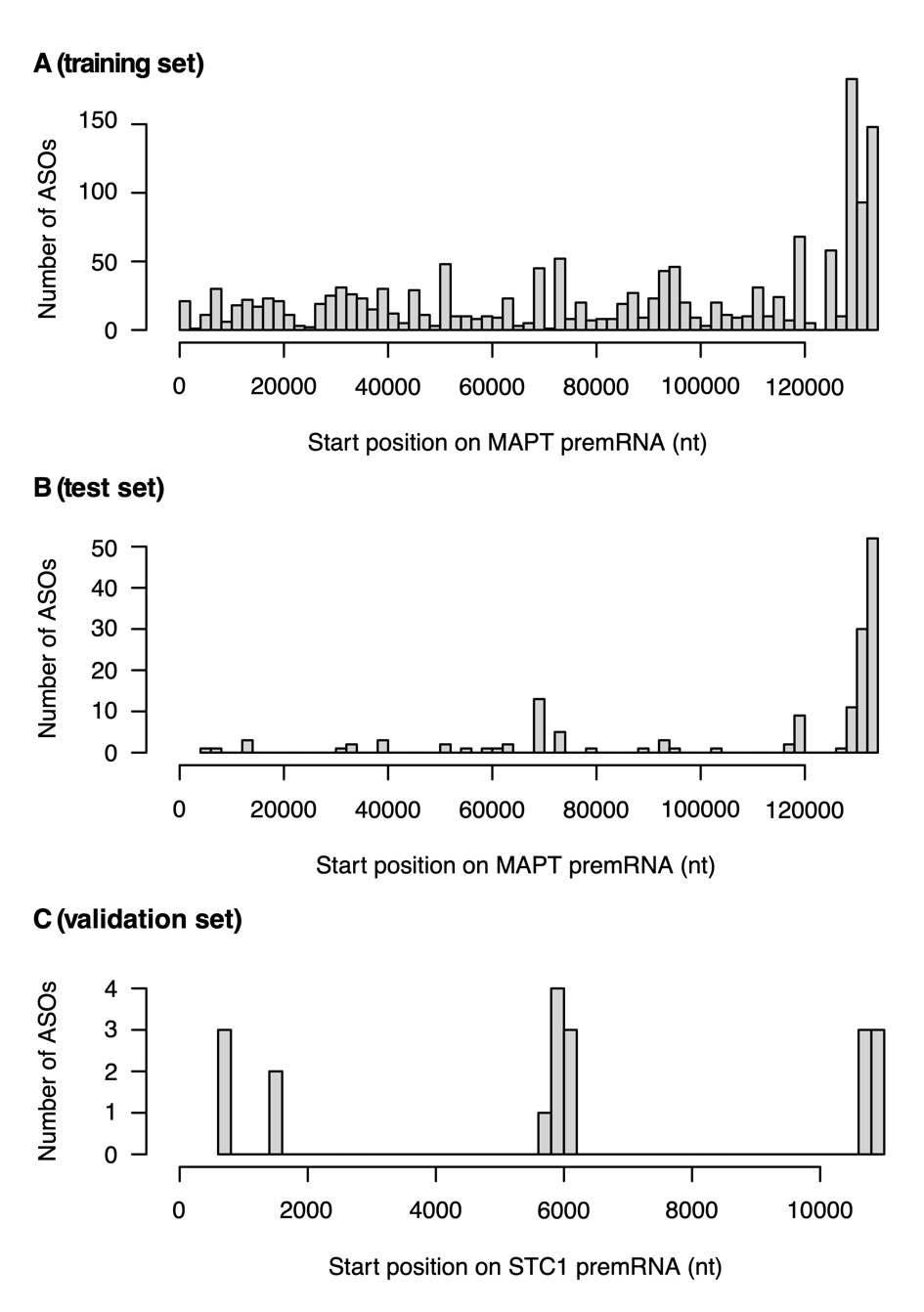


**Figure S5** *Distribution of ASO target regions across pre-mRNA* **A)** For the 1645 ASOs in the training set. This covers a total of 7063 nts distributed across 313 nonoverlapping regions on the MAPT pre-mRNA. **B)** For the 148 ASOs in the test set. This covers a total of 882 nts distributed across 44 nonoverlapping regions on the MAPT pre-mRNA. Out of these, 813 nts are overlapping with the training set. **C)** For the 19 ASOs in the validation set. This covers a total of 147 nts distributed across 7 nonoverlapping regions on the STC1 pre-mRNA.
