# Supplementary material for: Acute Neurotoxicity of Antisense Oligonucleotides After Intracerebroventricular Injection Into Mouse Brain Can Be Predicted from Sequence Features
Source: Nucleic Acid Ther. 2022 Jun 1;32(3):151–62. doi: 10.1089/nat.2021.0071 (PMC9221153; doi:10.1089/nat.2021.0071)
Supplement: Supplemental data [file Suppl_FigureS6.docx]

| 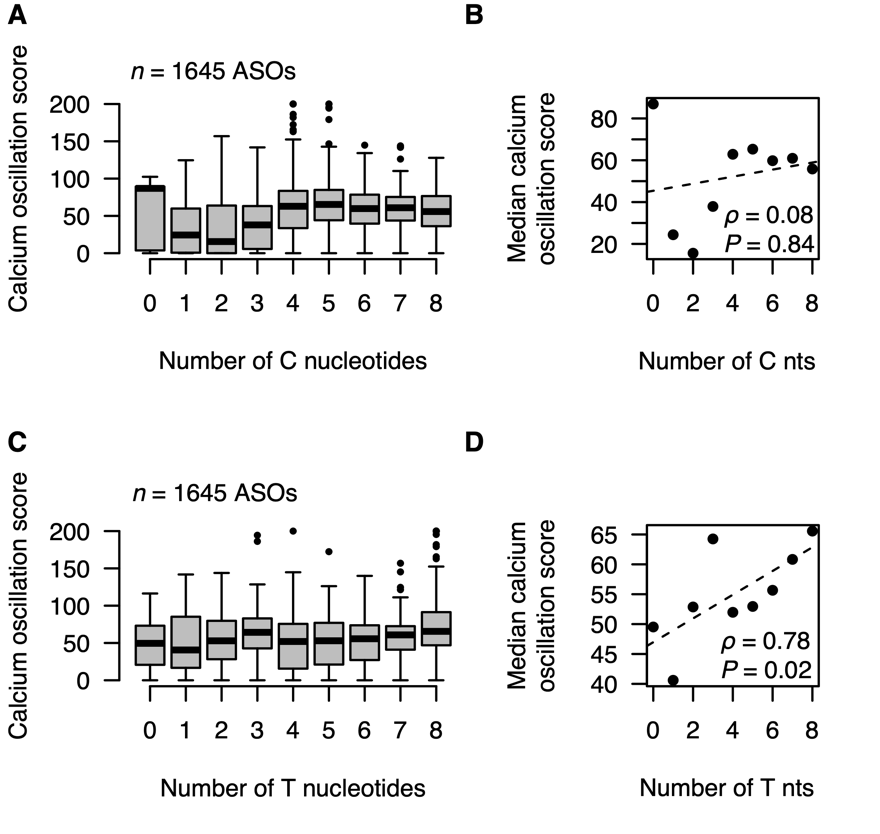 |
| --- |
| **Figure S6** *Associations between C/T nucleotide counts and calcium oscillation scores* **A)** Boxplots showing distributions of calcium oscillation scores for ASOs grouped based on the number of cytosine (C) nucleotides. **B)** Scatterplot of median calcium oscillation scores for ASOs with a given number of C nucleotides. Nonparametric correlation coefficient calculated as Spearman's rank correlation (*⍴)* with test for significance (*P*) using an ﻿asymptotic approximation of the Student's *t*-distribution. Dashed trend line calculated by linear least-square fitting. **C)** Boxplots showing distributions of calcium oscillation scores for ASOs grouped based on the number of thymine (T) nucleotides. **D)** As B) but for T nucleotides. |
