# Supplementary material for: Acute Neurotoxicity of Antisense Oligonucleotides After Intracerebroventricular Injection Into Mouse Brain Can Be Predicted from Sequence Features
Source: Nucleic Acid Ther. 2022 Jun 1;32(3):151–62. doi: 10.1089/nat.2021.0071 (PMC9221153; doi:10.1089/nat.2021.0071)
Supplement: Supplemental data [file Suppl_FigureS7.docx]

| 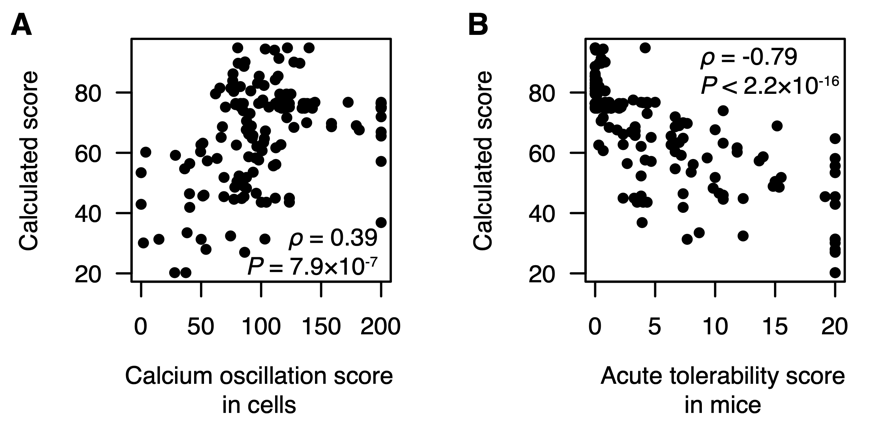 |
| --- |
| **Figure S7** *Association between acute tolerability and reductions in calculated score* **A)** Scatterplot of calcium oscillation scores evaluated in cells and calculated scores (equation 1) for *n* = 148 ASOs. Nonparametric correlation coefficient calculated as Spearman's rank correlation (*⍴)* with test for significance (*P*) using an ﻿asymptotic approximation to the Student's t-distribution. **B)** Scatterplot of acute tolerability scores evaluated in mice and calculated scores (equation 1) for *n* = 148 ASOs. |
