# Supplementary material for: Acute Neurotoxicity of Antisense Oligonucleotides After Intracerebroventricular Injection Into Mouse Brain Can Be Predicted from Sequence Features
Source: Nucleic Acid Ther. 2022 Jun 1;32(3):151–62. doi: 10.1089/nat.2021.0071 (PMC9221153; doi:10.1089/nat.2021.0071)
Supplement: Supplemental data [file Suppl_FigureS8.docx]

| 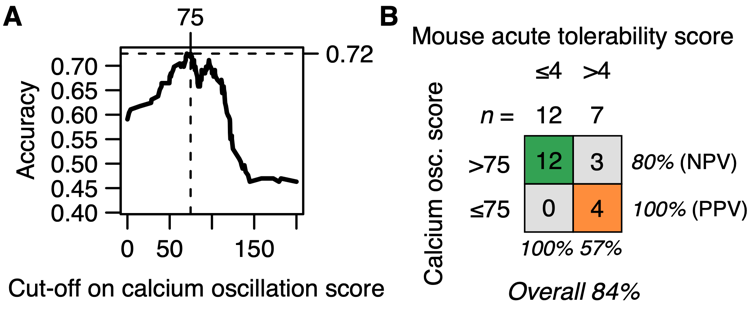 |
| --- |
| **Figure S8** *Classification performance of calcium oscillation scores evaluated in cells* **A)** Accuracy of the classification of ASOs in the test set as a function of the cut-off chosen for the measured calcium oscillation scores. Vertical dashed line indicates cut-off score resulting in maximal accuracy. **B)** Classification performance in the validation set using measured calcium oscillation scores with optimal cut-off score at 75. NPV, negative predictive value. PPV, positive predictive value. |
